# Review: An urgent need for research on factors impacting adherence to and retention in care among HIV-positive youth and adolescents from key populations

**DOI:** 10.7448/IAS.18.2.19393

**Published:** 2015-02-26

**Authors:** Priya Lall, Sin How Lim, Norliana Khairuddin, Adeeba Kamarulzaman

**Affiliations:** Centre of Excellence for Research in AIDS (CERiA), Department of Medicine, Faculty of Medicine, University of Malaya, Kuala Lumpur, Malaysia

**Keywords:** human immunodeficiency virus, young key populations, adherence, retention in HIV care, antiretroviral therapy

## Abstract

**Introduction:**

The 50% increase in HIV-related deaths in youth and adolescents (aged 10–24) from 2005 to 2012 highlights the need to improve HIV treatment and care in this population, including treatment adherence and retention. Youth and adolescents from key populations or young key populations (YKP) in particular are highly stigmatized and may face additional barrier(s) in adhering to HIV treatment and services. We reviewed the current knowledge on treatment adherence and retention in HIV care among YKP to identify gaps in the literature and suggest future directions to improve HIV care for YKP.

**Methods:**

We conducted a comprehensive literature search for YKP and their adherence to antiretroviral therapy (ART) and retention in HIV care on PsycInfo (Ovid), PubMed and Google Scholar using combinations of the keywords HIV/AIDS, ART, adolescents, young adults, adherence (or compliance), retention, men who have sex with men, transgender, injection drug users, people who inject drugs and prisoners. We included empirical studies on key populations defined by WHO; included the terms youth and adolescents and/or aged between 10 and 24; examined adherence to or retention in HIV care; and published in English-language journals. All articles were coded using NVivo.

**Results and discussion:**

The systematic search yielded 10 articles on YKP and 16 articles on behaviourally infected youth and adolescents from 1999 to 2014. We found no studies reporting on youth and adolescents identified as sex workers, transgender people and prisoners. From existing literature, adherence to ART was reported to be influenced by age, access to healthcare, the burden of multiple vulnerabilities, policy involving risk behaviours and mental health. A combination of two or more of these factors negatively impacted adherence to ART among YKP. Collectively, these studies demonstrated that future programmes need to be tailored specifically to YKP to ensure adherence.

**Conclusions:**

There is an urgent need for more systematic research in YKP. Current limited evidence suggests that healthcare delivery should be tailored to the unique needs of YKP. Thus, research on YKP could be used to inform future interventions to improve access to treatment and management of co-morbidities related to HIV, to ease the transition from paediatric to adult care and to increase uptake of secondary prevention methods.

## Introduction

According to WHO, youth and adolescents have become increasingly vulnerable to HIV infection [1]. In 2012, it was estimated that of the 35.3 million people living with HIV (PLHIV) globally, 5 million were aged 10–24. In the same year, over a third of new HIV-positive cases occurred among these age groups. During 2005–2012, while the global number of HIV-related deaths fell by 30%, the corresponding number among youth and adolescents increased by 50% [1]. The factors contributing to the higher mortality among HIV-positive youth and adolescents were the lack of awareness of sero-status [2–4], poor linkages between testing and treatment services [5], difficulty in retention in care [6] and lack of adherence to antiretroviral therapy (ART) regimes [7,8].

Youth and adolescents living with HIV can be broadly categorized into two groups according to routes of transmission: (1) infection at birth, that is, perinatally infected youth and adolescents (PIY), and (2) acquired by high-risk behaviours through injecting drug use and/or having condomless sex, that is, behaviourally infected youth and adolescents (BIY). It is important to note that adolescence can act as a transitional phase towards adulthood in which drug use and sexual experimentation are initiated, thus increasing the risk of contracting HIV [9,10]. For example, a national survey of high school students in the United States highlighted that 40.9% of adolescents had not used a condom in their last sexual encounter [11].

The WHO guidelines on testing and treatment of youth and adolescents aged 10–24 has identified men who have sex with men (MSM), transgender persons, people who inject drugs (PWID), sex workers and prisoners as young key populations (YKP). The term YKP recognizes that people belonging to these groups are at heightened risk of contracting HIV due to specific behaviours and social and legal environments which curtail their ability to protect themselves [7,12].

Currently little is known about how YKP engage with healthcare services while managing dual stigmas related to their HIV status as well as belonging to a marginalized section of the population. In order to understand these issues, it is useful to learn from the broader literature on youth and adolescents living with HIV in general. Research on PIY found a unique set of individual and environmental-level barriers to adherence and retention in HIV-related healthcare services. On the individual level, psychosocial barriers, such as depression and anxiety, have consistently been found to have an adverse effect on adherence of PIY to ART [13–15]. A cohort study of perinatally infected children and adolescents on ART in the United States found that depression or anxiety was predictive of non-adherence [16]. Meanwhile, on the environmental level, stigma [14], social or familial support [17] and socioeconomic status [15] can impact usage of healthcare services by youth and adolescents. People belonging to YKP can be stigmatized for their engagement in risk behaviours. Some HIV-positive YKP may, as a consequence, experience heightened socioeconomic and cultural barriers to accessing services owing to the fact that their already stigmatized status leaves them with few social or financial resources [9].

Previous reviews on adherence and retention in care in youth and adolescents have mostly focused on those who contracted HIV perinatally [13,18]. Although these reviews provide useful guidance on what types of individual and environmental-level barriers may affect HIV-positive YKP, individuals belonging to this group may have a slightly different set of needs to those who contracted it perinatally. For example, YKP who contracted HIV through injecting drug use could be in need of methadone treatment in addition to ART [19]. This literature review thus identified previous research on adherence to ART and retention in HIV-related care in YKP, discussed the current knowledge of individual and environmental-level barriers and facilitators to usage of healthcare services among these individuals, and suggested future directions in research to fill the gaps of knowledge and services in order to improve adherence to and retention in HIV care in these vulnerable populations.

## Methods

A comprehensive search for adherence to ART and retention in HIV care in YKP was conducted on PsycInfo (Ovid), PubMed and Google Scholar using combinations of the keywords HIV/AIDS, antiretroviral therapy, adolescents, young adults, adherence (or compliance), retention, MSM, transgender, injection drug users, PWID and prisoners. In addition, bibliographies of relevant articles were reviewed for supplementary studies. The inclusion criteria of the current review are empirical studies that (1) included key populations defined by WHO; (2) included the terms youth and adolescents or aged between 10 and 24; (3) examined adherence to or retention in HIV care; and (4) published in English-language journals. There were studies on adherence to and retention in HIV care among participants with wide age range but data were not disaggregated by age and therefore findings could not be inferred on the younger age group (10–24 years). These studies were excluded from the review. All articles were coded using NVivo. The systematic search yielded 26 articles dating from 1999 to 2014.

## Results and discussions

Our literature search yielded 26 studies overall, 20 of which were conducted in the United States (Table 1). Sixteen of these studies examined the adherence behaviours of BIY, where HIV was acquired through sexual risk behaviour or injecting drug use [20–31,33,34,39,42,43]. Seven other studies focused specifically on the treatment needs of HIV-positive young MSM (YMSM) belonging to ethnic minorities [32,36–38,40,41,44]. Finally, only two studies specifically assessed the adherence of young HIV-positive PWID [19,35]. Belzer *et al*. [42] focused on both BIY in general and YMSM.

**Table 1:** Studies conducted between the years 1999 and 2014 on adherence to ART and retention in care among BIY and YKP

Publication (first author, year)	Location (cities, country)	Study populations	Age (mean, range)	Sample size (HIV-positive)	Measurement of adherence to and/or retention in HIV care	Method	Intervention	Main findings
Belzer *et al*. 1999 [20]	Los Angeles, USA	BIY	15–24	31	Self-reported adherence	Quantitative (survey)	No	Medication adherence most significantly correlates with stability of living conditions in BIY.
Martinez *et al*. 2000 [21]	Cook county, Illinois, USA	BIY	13–21	25	Self-reported adherence	Quantitative (retrospective analysis of medical charts)	No	61% of subjects reported >90% compliance with their medications in the previous 90 days. 5 of 10 substance abusing subjects reported adherence to ART.
Murphy *et al*. 2001 [22]	13 cities in USA	BIY	12–19	161	Self-reported adherence, psychological theory to measure anxiety, social support and depression. Viral load (VL).	Quantitative (cohort study)	No	Higher levels of adherence associated with decreased depression, a strong association between adherence and reduced VL.
Rogers *et al*. 2001 [23]	USA	BIY	N/A^a^	288	Viral load and CD4 count	Quantitative (evaluation)	Yes	Only 18 of 288 participants received full TREAT programme, which led to adherence with ART.
Murphy *et al*. 2003 [24]	13 cities in USA	BIY	12–19	114	Self-reported adherence and viral load (HIV-1 RNA level in plasma)	Quantitative (survey)	No	Viral load was significantly associated with self-report of adherence to ART. Only 28.3% of adolescents reported taking all of their prescribed antiretroviral medications in the previous month.
Flynn *et al*. 2004 [25]	28 sites in the US and Puerto Rico in USA	BIY	8–22	120	Self-reported adherence and viral load	Quantitative (cohort study)	No	Adherence to ART was the only predictor of achieving undetectable virus loads.
Murphy *et al*. 2005 [26]	13 cities in USA	BIY	18.4, 12–18	231	Self-reported adherence, behavioural factors associated with adherence and viral load	Quantitative (survey)	No	Adolescents in the later HIV disease stage were less likely to be adherent compared with those in the earlier disease stage. Less alcohol use and being in school were associated with adherence by adolescents on weekends and over the preceding month.
Puccio *et al*. 2006 [27]	Los Angeles, USA	BIY	16–24	81	Self-reported adherence	Quantitative (pilot intervention study)	Yes	Most participants found the calls to be helpful and the level of intrusion into their daily lives acceptable. Using cell phone reminders to assist patients does not require an extensive amount of daily staff time.
Naar-King *et al*. 2006 [28]	USA	BIY	16–24	24	Self-reported adherence, self-efficacy, social support, psychological distress	Quantitative (survey)	No	Self-efficacy and psychological distress were significantly correlated with adherence but social support was not. Social support specific to taking medications was correlated with self-efficacy.
Rao *et al*. 2007 [29]	Chicago, USA	BIY	17–25	25	Self-reported adherence	Qualitative	No	Half of respondents indicated that they skipped doses because they feared family or friends would discover their status, suggesting that HIV stigma impacts treatment for youth on several levels, from the accuracy of communication with medical providers to medication adherence, subsequent health outcomes and the emergence of treatment-resistant strains.
Rudy *et al*. 2009 [30]	USA	BIY and blood products. Separate sexual abuse category	12–24	396	Survey instrument to measure adherence and outcome expectancy of adherence	Quantitative (observational study)	No	Non-adherence influenced by not having healthcare insurance, dropped out of school, homelessness and/or spent time in detention facility.
Garvie *et al*. 2010 [31]	Mid-southern USA	BIY, blood transfusion and unknown	16–24	60	Routine pharmacy pill count and self-reported. CD4 and VL.	Quantitative (survey)	No	The first study to measure adherence measurement based on both CD4 and VL. Non-adherence was related to off-schedule dosing.
Magnus *et al*. 2010 [32]	Bronx, Chapel Hill, Chicago, Detroit, Houston, Los Angeles, Oakland, Rochester, USA	AA, Latino YMSM	16–24	224	Retention defined as programme visits every three months	Quantitative (cohort study)	No	Retention associated with <21 years old, history of depression, receipt of programme services, feeling respected at clinic.
Comulada *et al*. 2003 [33]	Los Angeles, USA	BIY	14–29	253	Self-reported adherence, health status, sexual behaviour, substance use and psychological measures	Quantitative (survey)	No	Almost all youth had been offered ART (84%); 77% had ever used it, 54% were currently using and 63% of users adhered to 90% of their medications. Compared to non-users, users were more likely to be female, Latino or AA.
Agwu *et al*. 2011 [34]	17 US Clinic sites	BIY	18–24	3127	Self-reported adherence and clinic visits	Quantitative (retrospective study)	No	Youth PLHIV less likely to report injecting drug use behaviour. They were less likely to initiate ART.
Tapp *et al*. 2011^b^ [19]	Vancouver, Canada	YPWID	<24	PWID <24 (n=24), N=545	Adherence measured by compliance to prescription refill	Quantitative (cohort study)	No	Younger age (<24), being female, daily heroin injection and daily cocaine injection were negatively associated with 95% adherence while methadone treatment was positively associated with adherence.
Hadland *et al*. 2012^b^ [35]	Vancouver, Canada	YPWID	Median=37.2, age was dichotomized at 29	545	Self-reported adherence, VL	Quantitative (cohort study)	No	Young adults (age <29) were less adherent and were less likely to achieve VL suppression.
Wohl *et al*. 2011 [36]	Los Angeles, USA	AA and Latino YMSM	18–24	61	Retention associated with number of intervention visits, prescription of ART	Quantitative (pilot intervention study)	Yes	Highlights the critical needs of HIV-positive AA and Latino YMSM and demonstrate that a clinic-based YCM can be effective in stabilizing hard-to-reach clients and retaining them in consistent HIV care.
Hightow-Weidman *et al*. 2011 [37]	North Carolina, USA	AA and Latino MSM	Mean age 21	81	Retention defined as 1 medical visit every four months	Quantitative (cohort study)	Yes	Interventions on adherence need to actively reach out to youth populations.
Bouris *et al*. 2013 [38]	Chicago, USA	AA YMSM and TG	16–29	94	Self-reported adherence, VL	Quantitative (RCT)	Yes	Supportive relationships promote retention in care.
Barnes *et al*. 2013 [39]	Baltimore, New York City, Washington, USA	BIY, PIY	13–21	166	Assessed HIV knowledge	Quantitative (survey)	Yes	BIY outperformed PIY on questions related to disease awareness.
Gillman *et al*. 2013 [40]	Houston, USA	AA YMSM	Mean 19.9	47	Retention in care defined as completion of physician visits 90 days after linkage to care	Quantitative (survey)	No	Greater conspiracy beliefs were associated with negative medication attitudes while trust in physicians was correlated with positive medication attitudes; conspiracy beliefs were not associated with poor linkage to care and retention.
Harper *et al*. 2013 [41]	14 cities in USA	YMSM (66% AA, 19% Latino)	Mean 21.5, range 16–24	200	Self-reported adherence to medical appointment in the past three months	Quantitative (survey)	No	Ethnic identity affirmation and HIV-positive identity were associated with significantly higher risk for missed appointments in the past three months.
Belzer *et al*. 2013 [42]	Los Angeles, Washington, New Orleans, Fort Lauderdale, San Francisco, USA	BIY, YMSM	15–24	37	Self-reported adherence (dichotomized at 90%), viral load data abstracted from medical record	Quantitative	Yes	Intervention of daily cell phone conversation with health care providers. Self-reported adherence was significantly higher in intervention group than in the control group.
Saberi *et al*. 2014 [43]	USA	BIY, PIY	12–24	1317	Self-reported adherence in the past seven days (dichotomized at 100%); plasma HIV RNA	Quantitative	No	Pillbox was the most endorsed adherence device. Using adherence devices was inversely associated with having undetectable viral load. BIY more likely to be gay, adherent to ART and never been to jail.
Hussen *et al*. 2014 [44]	Atlanta, USA	YMSM	13–24	20	Self-reported adherence	Qualitative	No	Successful transition to adulthood and optimal ART adherence were inextricably linked. Detrimental impact of HIV on development was moderated by the degree of physical illness at diagnosis.

a Only specify participants as from REACH project

b these two studies were conducted on the same cohort.

AA=African American; ART=antiretroviral therapy; BIY=behaviourally infected youth and adolescents including sexual behaviour and injecting drug use; HAART=highly active antiretroviral therapy; HIV=human immunodeficiency virus; PIY=perinatally infected youth and adolescents; RCT=randomized control trial; REACH=Reaching for Excellence in Adolescents Care and Health; TG=transgender; TREAT=Therapeutic Regimens Enhancing Adherence in Teens; YCM=youth-focused case management; YMSM=young men having sex with men; YPWID=young people who inject drugs.

Research that specifically focused on adherence to ART regimes in HIV-positive youth and adolescents was relatively sparse because many clinical studies on treatment classified children and young adults into the age groups of around 0–14 and 15–24 [45,46], which overlaps the WHO definition of youth and adolescent of 10–24 years. These studies may fail to uncover factors affecting adherence which would be unique to HIV-positive adolescents (age 10–18) and young adults (age 19–24), simply due to different age categorization. There was even more of a dearth of literature on YKP, partly because it is more challenging to recruit HIV-positive young sexual minorities, sex workers, PWID and prisoners into research studies.

The literature search, thus, revealed that current knowledge on adherence to ART and retention in care in YKP was limited as research was heavily concentrated in the United States and focused on key populations which are of concern to that particular setting, including YMSM of ethnic minorities. To the best of our knowledge, there were no peer-reviewed articles that focused specifically on the treatment needs of young female sex workers, transgender youth and adolescents, and young offenders. There were, consequently, glaring gaps in the literature, as there appeared to be little to no research on adherence in YKP in developing countries, where most PLHIV live [46]. Furthermore, we were unable to find any studies that explored possible gender determinants of ART adherence, although 16 studies from the 26 studies reviewed here did involve female subjects.

The following section categorizes studies according to BIY in general and the different categories of YKP as per WHO classifications. We focus on individual and environmental factors related to adherence to ART and retention in care which were deemed as unique to each group. The final section of the results explores studies of interventions addressing the treatment needs of YKP.

### Research on BIY

Research on ART adherence among BIY did not focus on one particular key population, which resulted in some studies not differentiating participants according to their route of transmission and risk groups. Nonetheless, these studies do provide a useful overview of types of factors which may affect retention in care in YKP and how their adherence behaviours may differ from those of PIY.

Nine studies on BIY which included young people who inject drugs (YPWID) recruited participants from the Reaching for Excellence in Adolescent Care and Health (REACH) project [22–26,30,34,42,43]. REACH was originally designed as an observational study of HIV-positive patients attending “adolescent-specific medical care centres” in the United States [22]. Participants between the ages of 12 and 18 who contracted HIV through high-risk behaviours, such as condomless sexual contact or injecting drug use, were intentionally sampled in order to compare their behaviours to that of HIV-negative adolescents who engaged in similar behaviours and PIY. These studies found that risky sexual behaviours or injecting drug use, self-efficacy and positive mental health outcomes were associated with greater ART adherence.

Studies sampling from REACH suggested that adherence to ART in YKP were affected by a combination of individual and environmental-level barriers. At the individual level, younger age and history of depression were significantly associated with failure to adhere to complex medical regimes [26]. At the environmental level, unstable housing conditions [30] and lack of attendance at school acted as barriers to adherence [34]. Although there was no association between the ownership of medical insurance and initiation of ART, it was found that the type of medical insurance could influence their usage of healthcare services [34]. Those who were in receipt of publicly funded insurance were more likely to discontinue their treatment than those who had no insurance or private medical coverage. There was a possibility that “the small prescriptions co-payment that may be associated with publicly funded insurance programmes … may potentially serve as an impediment to … (art) continuation”, suggesting that participants with few financial resources may face many more difficulties in adhering to treatment [34], p. 6].

These studies also indicated that YKP who experienced a combination of both individual and environmental level barriers simultaneously may experience heightened difficulties in adhering to treatment. Rudy *et al*. [30] statistically tested the impact that the amount and type of barriers including not having healthcare insurance, having dropped out of school, homelessness and/or having spent time in detention facilities), as well as the existence of a mental disorder, had on adherence. It was found that 73% of participants with no barriers were adherent in comparison to 62% of those experiencing one barrier and 40% of those who reported two or more barriers. Moreover, 69% of respondents who had a low level of self-efficacy, a mental disorder and experienced at least one structural barrier were non-adherent.

Some HIV-positive youth and adolescents may attempt to mitigate the impact of individual and environmental level barriers on to adherence through actions which are not always beneficial towards their overall treatment. A focus group study of HIV-positive BIY on how they managed their HIV diagnosis found that many participants would conceal their sero-status from family and friends in order to avoid being stigmatized [29]. This resulted in half of the respondents skipping doses due to the fear that others would learn of their condition.

A few studies did, however, demonstrate that BIY were able to overcome environmental and individual level barriers in order to be more adherent to ART than their perinatally infected counterparts [31]. In a multi-clinic survey of HIV-positive youth and adolescents who were prescribed ART, Saberi *et al*. [43] found that full adherence was correlated with the behavioural route of infection, MSM behaviour, never being jailed and not using alcohol or illegal drugs]. It is possible that BIY may have more knowledge and awareness of their condition [39] than PIY due to the fact that they are expected to take charge of their treatment in the absence of family and social networks [17].

Although research on BIY provided a useful overview on individual and environmental level barriers which could potentially impact adherence to ART and retention in healthcare in YKP, very few studies distinguished between behavioural routes of transmission among their participants [26,31,33,34]. This resulted in different groups of YKP with varying treatment needs, such as YPWID and YMSM, being included into one category.

The other methodological issue was that many studies tested respondents’ socio-demographic characteristics against outcome variables without having *a priori* theory on how these factors may impact respondent's adherence to treatment [21,27,31,34]. Moreover, these studies often failed to include measurements of factors which could impact adherence and retention in care for youth and adolescents in general, let alone those belonging to key populations, such as their transition from child to adult healthcare services [44].

### Research on YMSM

Our literature search revealed only seven peer-reviewed publications yielded from research studies specifically on YMSM, most of which were conducted in the United States [32,36–38,40,41,44]. Six of these studies employ quantitative methods [36–38,40,41,44] , such as randomized control trials and surveys, to examine factors which could impact adherence to treatment regimes in YMSM, including age, ethnicity and sexual identity, while one study uses qualitative research methods [44].

In contrast to studies on treatment behaviours of BIY, research on African American YMSM was strongly informed by theories that took into account factors which were particular to their age, ethnicity and sexual identity. As a consequence, these studies were able to explore at length factors which could be unique to this particular YKP. One study examined respondents’ “conspiracy beliefs” in relation to HIV, namely the beliefs that the government was involved in the spread of HIV, had highlighted that many African Americans held these attitudes [40]. It was found that participants with greater “conspiracy beliefs” also had negative attitudes towards medication. None of these conspiracy beliefs, however, were correlated with CD4 counts at diagnosis, nor linkage and retention in care in the study, which suggested that participants were still willing to use treatment even if they did not fully trust their doctors.

As these studies incorporated theories and measurements which were relevant to YMSM, they were able to demonstrate that this population suffered from intersecting vulnerabilities due to increased stigma from belonging to three marginalized populations: ethnic minority, MSM, and HIV-positive. In a survey on HIV-positive YMSM of African American and Latino origins from Chicago, participants who held negative attitudes towards being gay and HIV-positive and strongly identified as belonging to an ethnic minority were more likely to miss clinical appointments [41]. These findings demonstrated the importance of understanding the development of multiple identities when treating YMSM from ethnic minorities.

In addition, YMSM belonging to ethnic minorities may experience environmental level barriers to adherence, such as poverty. For instance, in an assessment of a youth-focused case management intervention targeting HIV-positive YMSM from ethnic minorities, over three quarters of participants were in urgent neeed of stable housing, nutritional support, drug rehabilitation and mental health services at baseline [36]. These barriers to adherence were mitigated through increased number of intervention visits, more hours in the intervention and prescription of ART.

Finally, these studies were also able to identitify possible facilitators to adherence to ART and retention in care. A qualitative study of experiences of living with HIV and adherence to ART among African American YMSM found that their treatment behaviours were influenced by the developmental goals that they created as part of transitioning towards adulthood [44]. Participants who were able to attain self sufficiency through the development of a positive gay and HIV-positive identity were better able to adhere to medication than those who viewed their condition and sexual orientation in negative terms. These findings indicated that future interventions may need to tailor care of HIV-positive YMSM to engage with their developmental needs as well as their negotiation of multiple identities.

### Research on YPWID

The literature search identified only two studies, both came from a cohort study that investigated adherence behaviours of YPWID in Canada between 1996 and 2008 [19,35]. These studies did not focus on the youth and adolescent population as defined by the WHO; however, they did investigate factors which could impact ART adherence and retention in care in this particular group. One of these studies used age categorization of young adult (18–29 years) which did not fit into the WHO definition (18–24 years) [35]. Furthermore, the study did not disaggregate the data by age group, thus the results could not be inferred to the 18–24 age group. In the other study, participants who were younger than 25 years, female, and not receiving methadone treatment displayed a lower likelihood of being adherent to 95% of medication [19]. These findings suggest that female YPWID may experience greater barriers to adherence than their male counterparts, suggesting a gender bias. Further, female adult PWID were revealed to be harder to identify and procure healthcare services as they secure drugs from their abusive male partners [47].

### Research on interventions targeted towards YKP

From the 26 articles covered here, only seven intervention studies either described the latest programmatic developments or assessed the efficacy of a particular intervention focusing on adherence to ART and retention in healthcare services in the United States in YKP [23,27,32,36–39,42]. Three of these targeted BIY [27,39,42] with two of these studies using mobile phone technology to aid and monitor adherence. The other four intervention studies were conducted on African American YMSM. A randomized control trial of a cell phone adherence support intervention was conducted among HIV-positive BIY in comparison to PIY, where participants were reminded through daily telephone contact to take their ART medication [42]. There appeared to be no differences in adherence behaviours between BIY and PIY. Self-reported adherence was found to be significantly higher among participants belonging to the intervention group than that of the control. There were also medium to large effect sizes on self-reported adherence and viral load during the course of the study. These results indicated that mobile phone technology could be harnessed to encourage youth and adolescents to adhere to medications.

The other four studies covered interventions that are designed to improve ART adherence among HIV-positive African American YMSM [32,36–38]. These programmes provided care which was directed to the unique and complex psychosocial and physical health needs of this particular YKP. The Los Angeles County Department of Public Health actively targeted African American YMSM through community-based outreach services which encouraged participants to visit clinics for counselling and testing [32]. HIV-positive African American YMSM were referred to a youth-focused case management intervention. A study team from the Special Projects of National Significance then assessed these beneficiaries and found that over the first two years of study, only 11% of beneficiaries missed appointments for unknown reasons. Other factors that were associated with retention were feeling respected by medical staff and being in receipt of programme services. These findings suggested that the “youth centred” nature of these programmes may increase retention in treatment as YMSM feel respected and are given access to other services which catered to their psychosocial needs.

A few interventions that focused on treatment of HIV-positive YMSM tried to increase retention in healthcare services through developing and nurturing social and medical networks which could assist YMSM in overcoming individual and environmental level barriers to attending facilities [37,38]. For instance, Project nGage attempted to harness existing social support networks of HIV-positive YMSM to identify “support confidants” who provided beneficiaries with psychosocial assistance [38]. In addition, the Strength Through Youth Livin’ Empowered intervention used social marketing campaigns to target the social and sexual networks of potential peer leaders, which were later cultivated to provide the basis of a medical support network for those who had been newly diagnosed as HIV-positive [37]. Over a three-year period, 81 men were diagnosed or re-engaged in healthcare services and the odds of patients attending clinic visits increased two fold.

In summary, there have been programmes which have attempted to cater to the unique needs of this population using innovative methods, such as mobile phone technology. Magnus *et al*. [32] collected data from eight clinics across the United States to assess such programmes, and demonstrated that it was possible to refine existing youth-centred programmes to increase retention in care in YKP through the development of networks of providers, peer support groups and community-based services. Unfortunately, many of these studies reported the findings of small-scale pilot interventions which often had small sample sizes and were underpowered, hence limiting the generalizability of the results to the wider population of YKP [36,42]. For example, in Puccio *et al*. [27], the evaluation of an intervention using mobile phone technology had a sample of only eight participants.

## Conclusions

As the paradigm of HIV prevention has shifted to treatment-as-prevention strategies, it is necessary to identify factors that promote adherence to and retention in care to antiretroviral regimens among HIV-positive youth and adolescents [13]. Our literature search identified only a handful of studies on adherence to and retention in ART among YKP; seven of them on YMSM and two on YPWID. We expanded the review to include 16 studies on BIY; some of these studies did not specify risk behaviours thus we cannot be confident as to whether these studies have included YKP. Nonetheless, studies on BIY do provide a useful overview of types of factors which may affect retention in care in YKP and how their adherence behaviours may differ from those of PIY.

Most studies focused on YKP that are relevant to the HIV epidemic in the United States and Canada, which were BIY, YMSM belonging to ethnic minorities and YPWID. We were unable to find any research on certain groups of YKP identified by the WHO, including female sex workers and transgendered youth; although, it has been reported that these populations are more susceptible to HIV infection due to sexual exploitation, poverty, violence and stigma [9,48], all of which have the potential to impede their access to HIV services should they contract HIV.

Research on BIY and YKP highlighted that they suffered from a combination of individual- and environmental-level barriers to adherence, as a result of intersecting vulnerabilities owing to the fact that they experience from multiple forms of oppression. In many studies, the bulk of respondents belonged to ethnic minorities who have been historically marginalized in a Western context, were sometimes isolated from their social and familial networks and experienced bouts of imprisonment and housing instability. It was noted by researchers that respondents’ adherence to medication was found not necessarily to be affected by the existence of barriers but rather by the intensity and nature of these barriers. It was found in a few studies that respondents who took recreational drugs, experienced depression and were unable to afford private medical insurance were less adherent than those who did not report these barriers [19,26,34,35].

A recent study demonstrated that YKP were able to overcome these barriers and in some cases display higher rates of adherence to ART than their perinatally infected counterparts [18]. The results of these studies broke down common stereotypes associated with YKP by demonstrating that many adopted a responsible stance towards their treatment as they had few social or familial networks to rely on. This finding also suggested some YKP demonstrated strength and resilience in coping with challenges in engaging in HIV medical care.

In contrast, there exist a worrying paucity of research on adherence in YKP in developing countries as their health behaviours can vary by area. A systematic meta-analysis of studies mostly assessing adherence to ART in PIY illustrated that respondents living in Asian (84%, 95% CI 77–91) and African (84%, 95% CI 79–89) countries displayed higher rates of adherence than those located in North America (53%, 95% CI 46–59) [18]. As none of the studies conducted in Africa or Asia recorded the route of transmission or specifically targeted YKP, it was difficult to know if there was a unique set of factors influencing their adherence to treatment.

Taking all these findings together, we conclude that among YKP, individual and environmental factors including access to psychosocial support, experience of stigma, access to social and behavioural support, and socioeconomic status are important determinants to adherence behaviours. Existing intervention studies suggest that mobile phone technology, social marketing and support for social network may improve adherence among YKP, particularly YMSM. More research on young female sex workers, young transgenders and young offenders is urgently needed. While these populations are hidden and difficult to access, research studies in the United States and Canada demonstrate that accessing these populations is possible through developing research networks between academic institutions and clinics that provide services to these populations. The contexts in which other YKP seeking treatment and engaging in HIV care continuum are likely to be different and each YKP will require culturally tailored interventions to promote retention in and adherence to ART.

Structural factors such as added stigma and discrimination, marginalization, lack of social and family support, and poverty would most likely be barriers that impede YKP from continuing HIV care and treatment. Policy guidelines thus must undergo a paradigm shift to focus specifically on YKP and their unique needs as opposed to their adult counterparts.
